# Trends in the psychedelic renaissance: applying artificial intelligence to measure media portrayal of psychedelic drugs in the 21st century

**DOI:** 10.1192/bjo.2025.10974

**Published:** 2026-02-12

**Authors:** David A. Bender, Harrison M. Dunn, Amanda Pekau, Arushi D. Mohite, Akila Anandarajah, Brendan D. Ross, Jacob Steinle, Suraj Shankar, Brandon Kiley, Sara Martin, Rishi Gonuguntla, Mia Stonov, Nithya Pippala, Rana Abdalla, Madison K. Stille, Juy Yusuf, Madeline Villalba, Gibson Werner, Anvi Divekar, Melinda Daniels-Tineo, Hannah Wang, Sophia Chertock, Sonali Sharma, Syed Ali Ahmed, Reetwan Bandyopadhyay, Jatin Sridhar, Medha Iyer, Adebusola Adeyemi, Kayla Smart, Umer Jalil, Zaryab Alam, Baris C. Ercal, David J. Hellerstein, Charles B. Nemeroff

**Affiliations:** Department of Psychiatry, University of Texas at Austin Dell Medical School, Austin, Texas, USA; Department of Psychiatry, Washington University School of Medicine, St. Louis, Missouri, USA; Washington University in St. Louis, St. Louis, Missouri, USA; BJC Healthcare, St. Louis, Missouri, USA; Icahn School of Medicine at Mount Sinai, New York, New York, USA; McGovern Medical School at UTHealth Houston, Houston, Texas, USA; University of Illinois at Urbana-Champaign, Champaign, Illinois, USA; University of the Incarnate Word School of Osteopathic Medicine, San Antonio, Texas, USA; Harvard T.H. Chan School of Public Health, Cambridge, Massachusetts, USA; University of Texas Rio Grande Valley, Edinburg, Texas, USA; Texas A&M College of Medicine, Bryan, Texas, USA; Columbia University Vagelos College of Physicians and Surgeons, New York, New York, USA; New York State Psychiatric Institute, New York, New York, USA

**Keywords:** Psychiatry and law, psychological treatments, psychopharmacology, philosophy, psychotherapy

## Abstract

**Background:**

The relationship between media portrayal of psychedelic drugs, scientific research and drug policy is an area of debate.

**Aims:**

To apply artificial intelligence technology to measure trends in media sentiment towards the therapeutic potential of psychedelic drugs.

**Method:**

Up to 300 of the most relevant articles from Google News searches for the term ‘psychedelics’ were sampled for each year from 2000 to 2025. A large language model, ChatGPT, evaluated subject matter and sentiment.

**Results:**

In total, 88.3% of screened URLs (3308 of 3747) were included in the analysis. The proportion of articles focusing on the therapeutic potential of psychedelics increased from 13.3% (26 of 198) from 2000 to 2009 to 85.3% (1254 of 1470) from 2020 to 2025. The average sentiment score from 2000 to 2025 for articles from all publications (*N* = 2168) was 78.5 ± 9.3 (mean ± s.d.) (possible range: 1–100). 1.3% (29 of 2168) of articles carried negative sentiment (<50) whereas 4.8% (103 of 2168) had extremely positive sentiment (≥90). Average sentiment reached a peak in 2020 (80.8 ± 7.0), and a statistically significant trough in sentiment was observed in 2024 relative to 2020–2023 (2020–2023, 79.2; 2024, 74.3, *P* < 0.00001, Mann–Whitney *U*-test). The proportion of negative-neutral articles (≤65) increased annually from a trough of 3.6% (8 of 267) in 2020 to a peak of 20.9% (43 of 253) in 2024. Artificial intelligence sentiment scores were correlated and concordant with average human rater scores (*r* = 0.88, concordance correlation coefficient 0.84).

**Conclusions:**

Although most 21st-century media coverage of psychedelic drugs has been positively framed, negative and neutral coverage has increased in frequency since 2020. Researchers, clinicians, regulators and policy-makers should be mindful of the complex relationship between media portrayals of psychedelics and the results of scientific research.

Since the discovery of the psychoactive effects of lysergic acid diethylamide (LSD) in 1943,^1^ a complex relationship has existed between researchers studying psychedelic drugs and the popular media. Early media coverage of psychedelic drugs in the mid-20th century often treated these drugs with openness and curiosity, with first-person accounts of drug experiences and interviews with intellectuals or celebrities included as subject matter. Once LSD became more widely used in recreational settings, media coverage trended towards greater emphasis on adverse effects and sensationalistic negative themes, such as unproven claims that LSD causes chromosomal damage and birth defects.^2^ Commentators at the time expressed concern about the deleterious effects of both unduly positive and negative media coverage on the progress of research into therapeutic applications of LSD.^3,4^ In the wake of increasing negative publicity and opposition from the US federal government, support for scientific research gradually declined before ceasing entirely.^5^

US research into psychedelic drugs began again in the 1990s, with interest in their therapeutic potential increasing dramatically over the past two decades.^6–8^ Four commercial entities studying classical psychedelics have received U.S. Food and Drug Administration (FDA) breakthrough therapy designations,^9–12^ billions of dollars have been invested into companies studying classical psychedelic drugs or novel chemical analogues,^13^ four states have legalised the use of psychedelic drugs within specific settings^14^ and US federal government agencies have begun funding research into the potential therapeutic applications of psychedelics.^15^ General popular interest has increased simultaneously,^16^ with the term ‘The Pollan Effect’ coined to describe the impact of positive media coverage of psychedelics on public perception.^17,18^

In 2022, several researchers characterised this broad surge in interest as a ‘psychedelic hype bubble’ driven by media and commercial interests, expressing concern that this perceived excess in positive sentiment could negatively impact scientific research.^19^ Media outlets are incentivised to attract collective attention,^20^ which may contribute to imbalanced discussions of the risks or benefits of psychedelic drugs. This in turn may promote extremes of opinion regarding their appropriate usage and processes of implementation.^2^

Various commentators consider the year 2024 to be a negative period for the advancement of the clinical application of psychedelic drugs,^21,22^ which included the rejection of the FDA application for the use of 3,4-methylenedioxymethamphetamine-assisted therapy (MDMA-AT) in the treatment of post-traumatic stress disorder (PTSD). Over the course of that year, several articles were published in major media outlets highlighting various criticisms of the perceived quality of psychedelic research,^22–25^ with other commentators suggesting that the rejection of MDMA-AT had resulted from bias.^26–28^ Within this evolving media landscape, understanding the relationships between media portrayal of psychedelics, the results of scientific research and political decision-making is especially important.

To our knowledge, two peer-reviewed publications have examined trends in media portrayal of psychedelic drugs in the 21st century, identifying various trends including increasing coverage and increasingly positive sentiment for certain media outlets.^29,30^ These studies have been limited to analysis of up to four newspapers and have not applied modern large language models (LLMs) to perform sentiment analysis at scale. This study employs ChatGPT to quantify trends in media portrayal of the therapeutic potential of psychedelic drugs during the 21st century.

## Method

### Data collection

Searches for the term ‘psychedelics’ at news.google.com were conducted within private browser windows between January and March 2025 for each calendar year 2000–2025. Every URL derived from each search was extracted (up to 300 per year) and screened by human raters, who determined whether the web page met broad inclusion criteria for constituting an analysable online media article, which included the following: having a date of publication, having adequate text to analyse, not being solely an advertisement, not being published in a peer-reviewed scientific journal and having a functioning URL link (Supplementary Materials, Appendix A available at https://doi.org/10.1192/bjo.2025.10974). If human raters were unsure whether a URL met inclusion criteria, that URL was referred to the first author for evaluation. We included articles from a broad variety of online media websites (e.g. major media outlets, academic news media, specialist medical media websites, advocacy websites) to minimise sampling bias. Included URLs were analysed by ChatGPT to determine whether their content focused on the therapeutic potential of psychedelics and, if so, to assign a sentiment score (range 1–100) regarding how positive the article is about the therapeutic potential of psychedelics (Supplementary Methods). A ChatGPT-4-based model was the default during the period of data collection. Total numbers of URLs for each year were determined by summing totals for Google searches confined to calendar years (Supplementary Methods).

### Human versus artificial intelligence comparisons

**Fig. 1:**
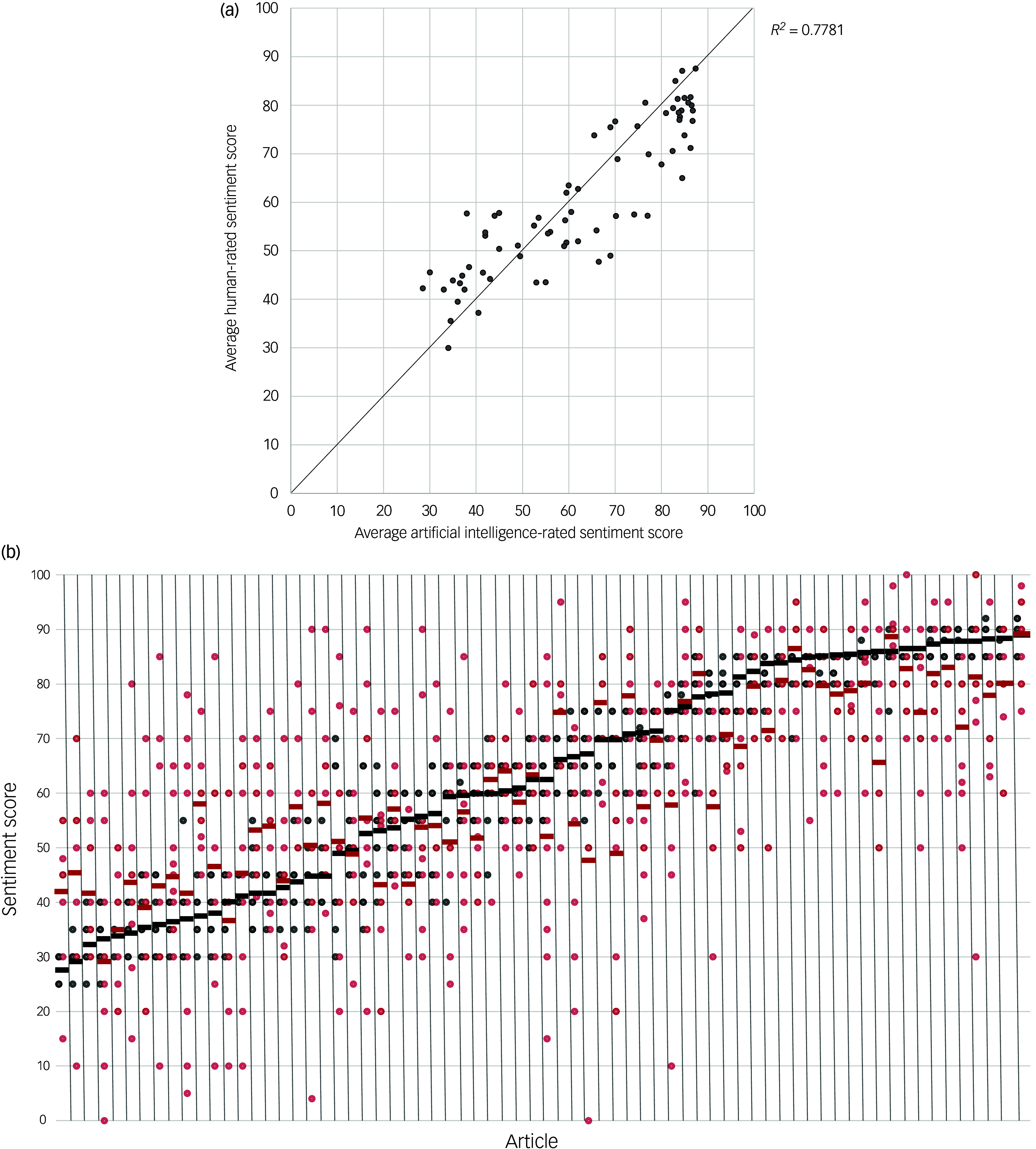
Comparison of the performance of artificial intelligence and human raters in measuring media sentiment towards psychedelic drugs. (a) Scatter plot comparing the average artificial intelligence sentiment score for 70 randomly sampled articles with the average sentiment score of human raters (9–10 raters per article). The plotted line represents the theoretical line of complete concordance between artificial intelligence and human ratings. (b) Plots of each individual sentiment score rating for human raters and repeated artificial intelligence iterations for all 70 articles. Each column on the *x* axis represents an individual article, with articles sorted left to right from lowest to highest average artificial intelligence rating. Black points denote individual artificial intelligence ratings and red points denote individual human ratings. The thickness of borders for individual points represents the number of ratings at a specific sentiment score, with thicker borders indicating more ratings at that score. Black and red bars represent average scores for artificial intelligence and human raters, respectively.

A random number-generator (Matlab 2024b, Mathworks) was used for random selection of approximately equal numbers of URLs from three tiers of articles distinguished by artificial intelligence-determined sentiment score (0–49, 50–74, 75–100), with 70 articles in total selected. These URLs were divided into 3 groups with approximately equal representation of sentiment score tiers, which were assigned to 3 subgroups of human raters (9–10 raters per group; Supplementary Materials, Appendix B). Human raters had varying educational levels (at minimum, high school graduate), and prior knowledge about psychedelic drugs was not a selection criterion. Each human rater was asked to independently score the sentiment of the articles provided using the identical prompt offered to the artificial intelligence (Supplementary Materials, Appendix C). Corresponding artificial intelligence scores for assigned articles were not provided to human raters. For each article scored by human raters, ten total repetitions of the data collection procedures (Supplementary Methods) were performed to collect ten artificial intelligence sentiment scores. Collected human rater and artificial intelligence sentiment scores were averaged for each article for the data-set shown in Fig. 1(a).

A random number-generator was used to select 15 articles from Google News judged by artificial intelligence to focus on the therapeutic potential of psychedelic drugs, and 15 articles that artificial intelligence had judged as not focusing on the therapeutic potential of psychedelic drugs. Human raters were asked to give a yes or no answer to the identical prompt provided to the artificial intelligence for these 30 articles (see Data collection). Human raters were not provided with the answer previously offered by the artificial intelligence. Articles were considered to have ambiguous content if the percentage of human raters answering the majority response consisted of between >50 and ≤70% of the rater population, or if equal numbers of human raters answered yes and no. For a given article, if no human majority occurred due to equal numbers of human raters answering yes and no (*n* = 1), artificial intelligence was by default assigned as agreeing with the majority of human raters.

### Statistical analysis

The Mann–Whitney *U*-test conducted in Matlab 2024b was used to compare sentiment score averages between different subpopulations of scores (sentiment in 2020–2023 versus 2024; human versus artificial intelligence sentiment scores).

Interrater reliability was calculated for subgroups of articles rated by the same group of human raters using the intraclass correlation coefficient (ICC) 2,*k*,^31^ with computations performed in Microsoft Excel. ICC was calculated for each article subgroup for both human raters and artificial intelligence scores derived from ten scoring iterations.

The concordance correlation coefficient between averages of artificial intelligence and human scores for individual sampled articles was calculated in accordance with established procedures,^32^ with computations performed in Microsoft Excel.

## Results

**Fig. 2:**
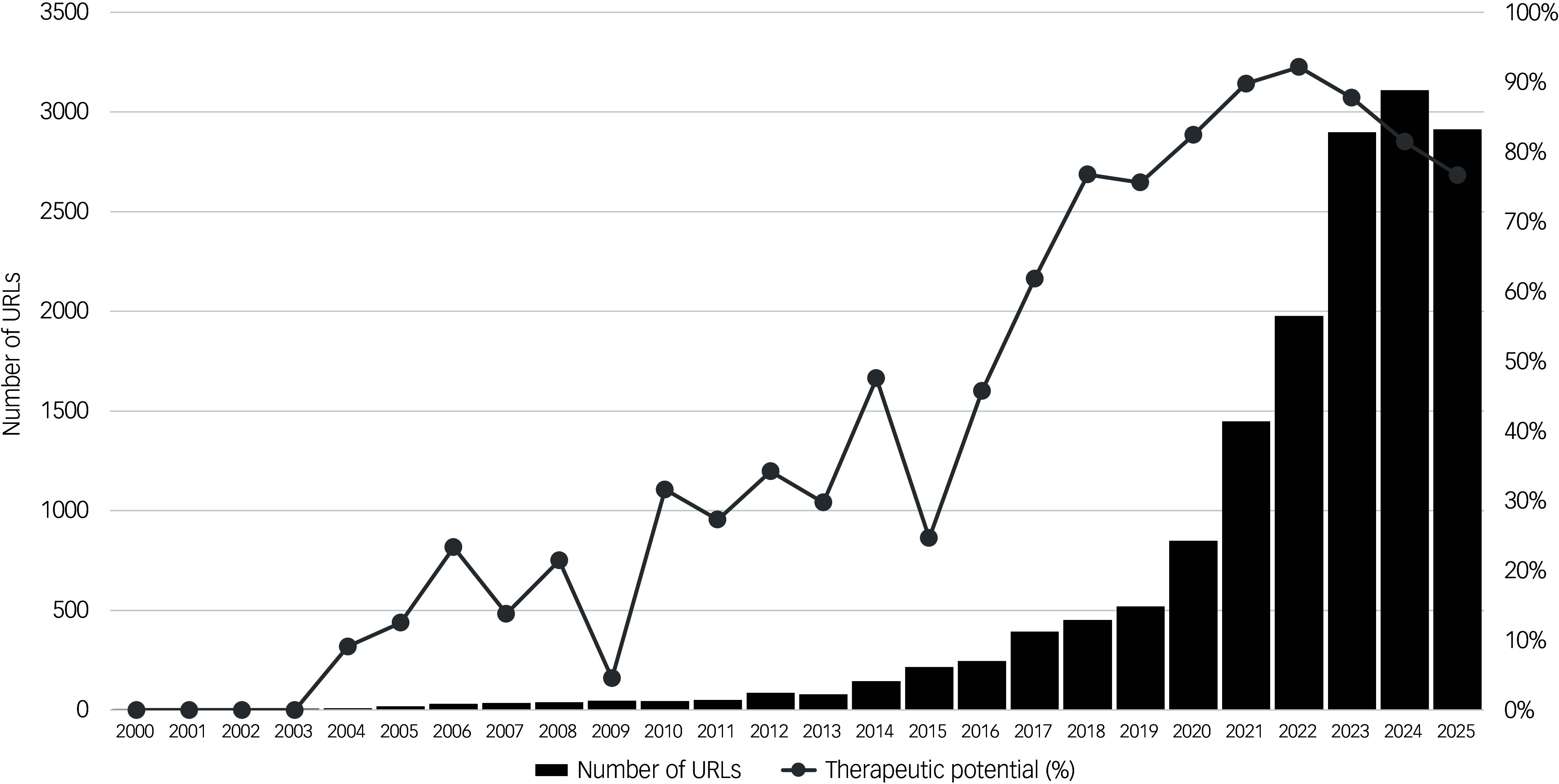
Changes in media coverage of psychedelic drugs in the 21st century. Black bars represent the total number of URLs appearing in Google News searches for the term ‘psychedelics’, confined to individual calendar years, with totals labelled on the left-hand *y* axis. The total for the year 2025 is a projection determined by multiplying the total URLs in January–March by 4. The line chart represents the percentage of analysed articles judged by artificial intelligence as primarily pertaining to the therapeutic potential of psychedelic drugs for each calendar year.

A total of 3747 URLs were screened, of which 88.3% (3308) were included in the analysis (Supplementary Materials, Appendix A). Of these articles, 65.5% (2168) were judged by artificial intelligence to primarily pertain to the therapeutic potential of psychedelic drugs. The proportion of media articles relating to psychedelics that focus on their therapeutic potential has markedly increased since 2000, with 13.3% (26 of 198) of articles from 2000 to 2009 focusing on this topic compared with 85.5% (1254 of 1470) from 2020 to 2025 (Fig. 2). The total number of URLs appearing within Google News for the search term ‘psychedelics’ increased from a minimum of 4 in 2000 to a maximum of 3108 in 2024, with a 501% increase in total numbers between 2019 and 2024 (520 to 3108) (Fig. 2). Similar trends were observed when articles were sampled from the Harvard Media Cloud online media database (Supplementary Materials, Appendix D).

Analysed articles were published on 1004 different websites (Supplementary Materials, Appendix E), with those published in the top 50 most heavily trafficked English-language media websites accounting for 19.6% (650 of 3308) of the data-set (Supplementary Materials, Appendix F). Individual websites with the most articles focusing on the therapeutic potential of psychedelics included VICE (62 articles), *The Guardian* (61 articles), Multidisciplinary Association for Psychedelic Studies (55 articles), *Business Insider* (53 articles) and *The New York Times* (48 articles) (Supplementary Materials, Appendix G).

Table 1 provides examples of analysed articles, alongside sentiment scores and rationales produced by artificial intelligence to offer context for quantitative analysis.

**Table 1:** Examples of artificial intelligence sentiment analysis. Artificial intelligence was asked to rate the positivity of individual articles about the therapeutic potential of psychedelic drugs, on a scale of 1–100, where 100 denotes very positive and 1 very negative. Example artificial intelligence responses are shown

Year	Title	Publication	Sentiment score	Rationale for score
2018	Ending America’s war on drugs would finally unleash the therapeutic potential of psychedelics	*TIME Magazine*	95	The article by Rick Doblin is very positive about the therapeutic potential of psychedelic drugs. It highlights the success of MDMA-assisted therapy for PTSD, criticises the barriers to research due to drug prohibition and advocates for the mainstream medical use of psychedelics. Given the strong endorsement and emphasis on benefits, I would rate it 95 out of 100 in terms of positivity.
2025	The evidence is clear: Nevada must move forward on psychedelic therapies	*The Nevada Independent*	95	This article presents a very positive perspective on the therapeutic potential of psychedelic drugs, particularly in addressing mental health issues such as PTSD, depression and substance use disorders. The authors, both of whom have personal experience with the mental health crisis, advocate for immediate action to implement psychedelic-assisted therapy in Nevada, emphasising the significant evidence supporting the safety and efficacy of substances including psilocybin, ibogaine and 5-MeO-DMT. The article conveys a sense of urgency, stressing the severe mental health crisis in Nevada and the need for bold legislative action. It highlights the promise of psychedelic therapies as a solution, coupled with a strong call for decriminalisation and the establishment of regulated programmes to ensure safe access. On a scale from 1 to 100, I would rate this article as 95. The tone is highly positive, focusing on the clear benefits of psychedelic therapy and the urgent need for change, with little scepticism or negativity.
2017	What it’s like to take psychedelics in small doses at breakfast	*NewScientist*	65	Based on the content you provided, the article seems somewhat neutral to mildly positive. Although it acknowledges the potential benefits of microdosing and personal experiences of people like Janet Lai Chang, it also highlights the lack of rigorous scientific evidence and the challenges associated with researching these substances due to their illegal status. The article presents both the positive aspects (e.g. reduced anxiety, increased openness) and the potential negative side-effects (e.g. confronting repressed emotions), offering a balanced view. I’d rate it around 65 out of 100, with a slightly positive tone due to the recognition of the benefits and personal testimonies, but tempered by the uncertainty and limitations highlighted.
2024	The biggest unknown in psychedelic therapy is not the psychedelics	*Vox*	55	On a scale of 1–100, I would rate the article at around 55. Although the article does acknowledge the therapeutic potential of MDMA and its potential to help people with PTSD, it presents significant concerns about the safety, scientific rigour and ethical issues surrounding the therapy practices used by Lykos Therapeutics. The article highlights issues with the therapy model, including accusations of cult-like behaviour, lack of rigorous scientific study on the therapy component and potential harms to participants. Thus, although the article doesn’t outright reject the idea of psychedelic therapy, it leans towards scepticism due to the concerns raised, making it moderately positive but with substantial caution.
2024	How psychedelic research got high on its own supply	*The New York Times*	40	The article expresses significant scepticism about the therapeutic potential of psychedelic drugs, especially MDMA, due to issues such as flawed clinical trials, ethical concerns and exaggerated claims by proponents. Although it acknowledges some positive possibilities, the tone leans more towards caution and criticism of the current state of research and its practices. On a scale of 1–100, I would rate the positivity of this article about the therapeutic potential of psychedelic drugs at around 40, reflecting its acknowledgment of potential benefits but emphasising concerns and shortcomings in the field.
2024	The weak science behind psychedelics	*The Atlantic*	35	Based on the article you provided, I would rate its positivity about the therapeutic potential of psychedelic drugs at around 35 on a scale of 1–100. The article provides an in-depth critique of the scientific research surrounding psychedelics, emphasising the weaknesses in methodology, ethical concerns, underreporting of adverse side-effects and the potential for overhyping the drugs’ effectiveness. Although it acknowledges that psychedelics show some promise in treating conditions like depression, PTSD and addiction, it stresses the need for more rigorous and unbiased research. The tone is cautionary rather than fully supportive, urging better evidence and caution in their use, particularly for vulnerable patients.

MDMA, 3,4-methylenedioxymethamphetamine; PTSD, post-traumatic stress disorder; 5-MeO-DMT, 5-methoxy-N,N-dimethyltryptamine.

**Fig. 3:**
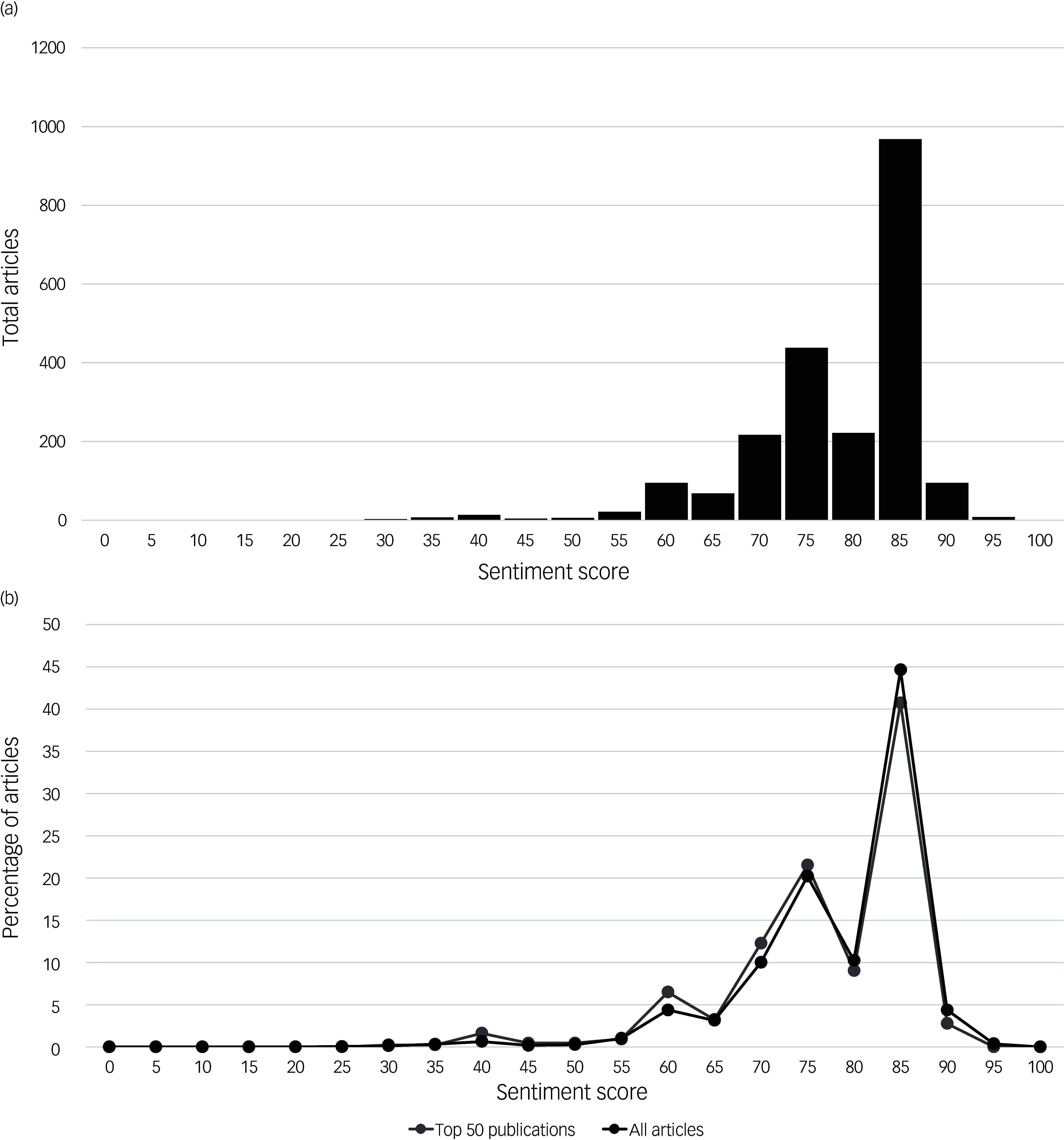
Media sentiment towards the therapeutic potential of psychedelic drugs in the 21st century. (a) Bar chart demonstrating the total number of articles with each sentiment score as determined by the large language model. (b) Line chart demonstrating the percentage of all articles with specific sentiment scores from article subgroups.

Overall, 21st-century media sentiment regarding the therapeutic potential of psychedelic drugs was heavily skewed towards positive portrayals (Fig. 3(a)), with 98.7% of articles having a score of 50 or greater (2139 of 2168) and 89.9% having a score of 70 or greater (1948 of 2168). Extreme positive portrayals (sentiment score ≥90) accounted for 4.8% (103 of 2168) of all articles, while negative portrayals (sentiment score <50) accounted for only 1.3% (29 of 2168). Similar results were found among the top 50 most trafficked English-language media websites (Fig. 3(b)), with an average sentiment score of 76.8 (*n* = 432) compared with 78.9 among websites outside the top 50 (*n* = 1736). For the the top 50 online media outlets with at least 5 articles pertaining to the therapeutic potential of psychedelic drugs in the data-set (*n* = 20), average sentiment ranged from a minimum of 67.0 (*Daily Mail*) to a maximum of 80.9 (CNN) (Supplementary Materials, Appendix G).

**Fig. 4:**
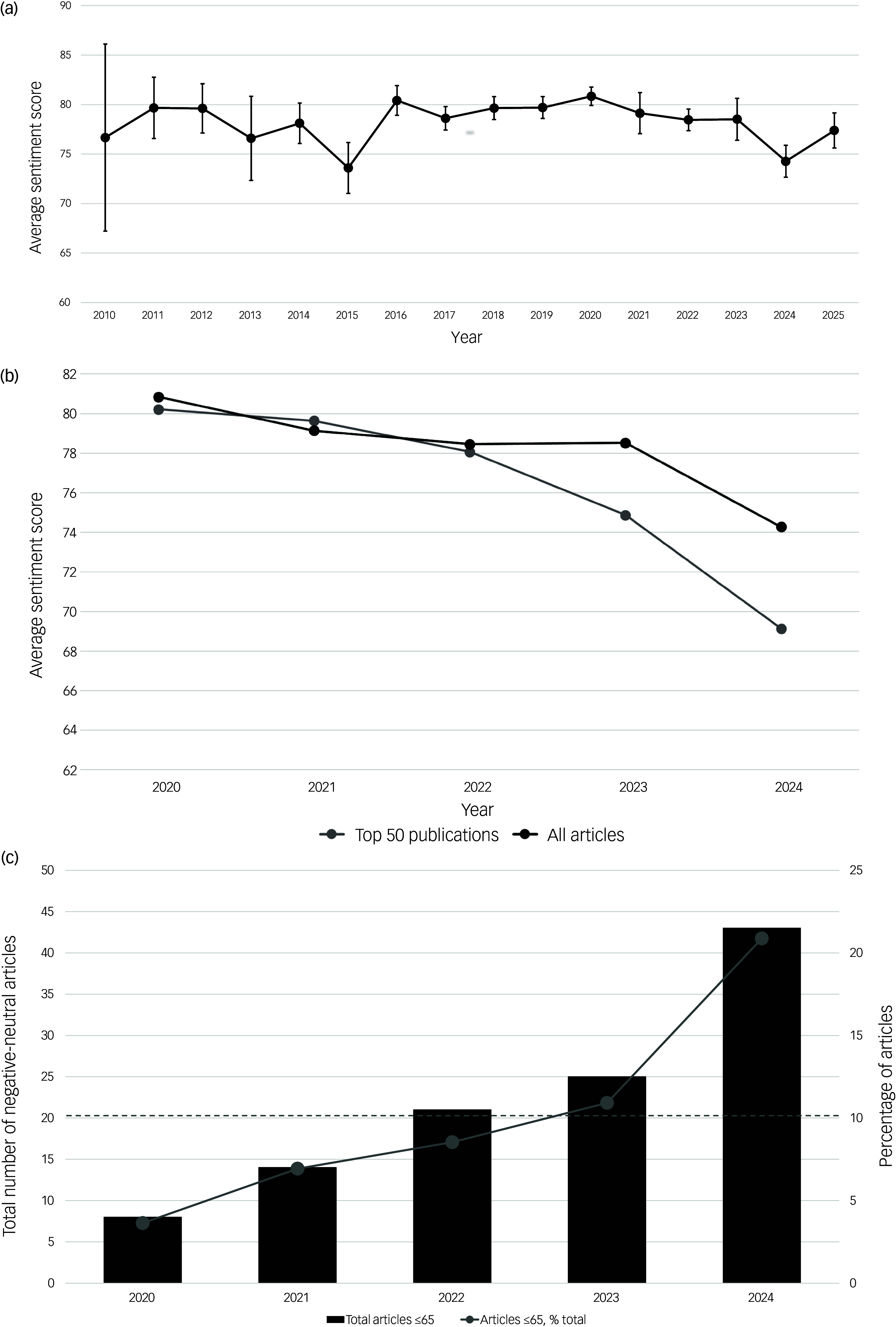
Temporal shifts in media sentiment towards the therapeutic potential of psychedelic drugs. (a) Average sentiment score for individual years from 2010 to 2025, with bars representing 95% confidence intervals. The year 2025 sampled articles from only 2 calendar months (January and February). (b) Average sentiment score during a period of decline in sentiment from 2020 to 2024, distinguishing between article subgroups. (c) Increases in the absolute numbers (black bars) and percentages of negative-neutral articles (grey line) (sentiment score ≤65) from 2020 to 2024, with the left-hand *y* axis indicating the total number of negative-neutral articles and the right-hand *y* axis indicating the percentage of articles. The dashed line indicates the percentage of negative-neutral articles within the entire data-set.

Average media sentiment from 2010 to 2025 is depicted in Fig. 4(a), ranging from a minimum of 73.6 in 2015 to a maximum of 80.8 in 2020. A statistically significant decline in sentiment occurred between 2020–2023 and 2024, the year in which the FDA application for MDMA-AT was not approved (2020–2023, 79.2; 2024, 74.3, *P* < 0.00001; Mann–Whitney *U*-test). Absolute declines in sentiment were greater in magnitude for the top 50 publications (Fig. 4(b)). Both the total number and proportion of analysed articles with negative-neutral sentiment (sentiment score ≤65) increased annually from a trough in 2020 until a peak in 2024, with negative-neutral articles accounting for only 3.6% of articles in 2020 compared with 20.9% in 2024 (Fig. 4(c)).

Among 70 randomly sampled articles from three sentiment score tiers (0–49, 50–74, 75–100) scored by human raters, average artificial intelligence sentiment scores were both strongly correlated, and often concordant with, average human scores (Pearson’s *r* = 0.88, 95% CI [0.81, 0.92]; *P* < 0.00001, complete concordance coefficient 0.84) (Fig. 1(a)). Correlations of artificial intelligence scores with individual human raters varied widely (minimum correlation, *r* = 0.28; maximum correlation, *r* = 0.93) (Supplementary Materials, Appendix H). There was no significant difference when comparing all human sentiment scores versus all artificial intelligence scores (Mann–Whitney *U*-test; average artificial intelligence score, 61.9; average human score, 60.8). Artificial intelligence sentiment scores were slightly higher than those of human raters for articles with positive sentiment (average 73.1 *v*. 67.6 for humans), and slightly lower for those with negative sentiment (average 39.1 *v*. 46.0 for humans).

Figure 1(b) shows all artificial intelligence and human sentiment scores for each sampled article. Artificial intelligence had high interrater reliability across repeated iterations of sentiment scoring (ICC 2,*k* for article subgroups 1, 2, 3 = 0.94, 0.93 and 0.93, respectively), while human raters had comparatively weak interrater reliability (ICC 2,*k* for article subgroups 1, 2, 3 = 0.56, 0.50 and 0.51, respectively) (Supplementary Materials, Appendix B).

The subject matter of 30 semi-randomly sampled articles was assessed by human raters regarding whether they primarily pertained to the therapeutic potential of psychedelic drugs. Majority human opinion concurred with artificial intelligence for 76.7% (23 of 30) of articles. Concurrence between human intelligence and artificial intelligence was higher for articles with unambiguous content, with artificial intelligence agreeing with the human majority for 89.5% (17 of 19) of these articles compared with 54.5% (6 of 11) of articles with ambiguous content (see Methods).

## Discussion

This study has several key results. As anticipated, our results indicate a dramatic increase in media interest in psychedelic drugs since the beginning of the 21st century, which has coincided with a subject matter shift towards their therapeutic potential. Although 21st-century media have been heavily skewed towards positive coverage of their therapeutic potential, this coverage has been only moderately positive on the study scale, with articles sampled from the top 50 media outlets carrying an average sentiment score of 76.8 out of 100. Although the average sentiment score has been moderately positive throughout the 21st century, it significantly declined in 2024 and the proportion of negative-neutral articles (sentiment ≤65) has been steadily increasing since reaching a trough of 3.6% in 2020.

The method of using a LLM to quantify sentiment was validated by comparison with 29 human raters. Artificial intelligence sentiment scores were highly correlated with average human scores, and often concordant (Fig. 1(a)). This suggests that the use of the LLM to measure sentiment, as employed in this study, is an overall close approximation of aggregated human intelligence performing the identical task. Moreover, the LLM was substantially more reliable in its measurements than were human raters. In principle, our general approach to sentiment analysis could be broadly utilised across other types of media studies and the social sciences.

Our study has several limitations. Analysed articles were sampled from the most relevant articles from each calendar year, as determined by the Google News search engine. Thus, indexing procedures from Google News will impact the sample and could have led to sampling bias. For example, Google News could be more likely to index positively framed articles due to increased reader engagement, although we did not find any relationship between article index rank and sentiment score within our data-set (Supplementary Materials, Appendix I). Google News is also unlikely to comprehensively index all possible media articles. While this is a socially important sample, given that Google is the most widely used search engine,^33^ the use of other approaches to sampling of online media articles could lead to distinct results. Our use of the search term ‘psychedelics’ – although this term is now commonly used to refer to this class of drugs, including by the National Institute on Drug Abuse^34^ – may have biased results towards positively framed articles relative to the use of other search terms such as ‘hallucinogen’. Moreover, the term psychedelic is broad and may refer to a variety of heterogeneous compounds in the context of a Google search (e.g. psilocybin, LSD, ketamine, MDMA). This limits our knowledge of which specific drugs with distinct effects are being evaluated in the data-set, and to what degree.

The underlying mechanics of how the LLM determines sentiment scores cannot be known at a granular level due to the complexity of the model, and there is a potential for misinterpretation associated with any limitations of the model. The use of other LLMs could produce different results. The use of the phrase ‘therapeutic potential’ in identifying article subject matter, which addresses the fact that psychedelic drugs do not have widely accepted therapeutic uses, may lead to articles that are negative about psychedelic drug use being excluded from sentiment analysis. Insofar as our data are viewed as a general proxy for media sentiment towards psychedelics, this could lead to an overestimation of average sentiment. However, that is unlikely to make a material impact on sentiment analysis for 2020–2025, given that 85.3% of these articles were deemed to focus on the therapeutic potential of psychedelics. Articles were sampled from only the first 2 months of 2025, which may have led to sampling biases unique to this calendar year. Increases in the sample size of human-rated articles would offer further precision for the determined correlation between human and artificial intelligence raters.

Our results have several implications that can inform ongoing dialogue about the societal value of psychedelic drugs, and the impact of media coverage on their perception. Prior commentators have expressed concerns about the negative effects of sensationalistic positive media coverage of psychedelic drugs.^19^ Although our results indicate a marked positive skew in media coverage, overall sentiment was consistent with only a moderately positive outlook towards the therapeutic potential of psychedelics. However, this persistently positive media sentiment probably played a role in the decision of voters and state legislators in several US states to legalise the use of psychedelic drugs despite a lack of federal approval for use within medical settings, which could have negative effects on public health. It may have also contributed to an increase in recreational use,^35^ which can also have deleterious effects.^36–39^ Elevated media sentiment could also affect the expectations of participants in clinical trials, which may impact subsequent experiences.

Sentiment regarding the therapeutic potential of psychedelics reached a peak in 2020 and declined until reaching a trough in 2024, consistent with the hypothesis that there has been a backlash resulting in part from excessively positive media appraisals.^19^ Several articles in major media outlets were published in 2024 offering comparatively sensationalistic negative characterisations of psychedelic research programmes.^22–24^ This shift coincided with the application for MDMA-AT for PTSD being rejected by the FDA, a decision which was criticised by various groups.^26–28,40,41^ These circumstances highlight the complex relationship between science and the media in the case of psychedelic drugs, in which media coverage and public opinion may have a greater impact on policy-making and regulatory processes than in other fields of research. Various commentators have emphasised that the scientific evaluation of psychedelic therapies has been held to uniquely stringent standards in regulatory settings relative to other approved and widely used psychiatric interventions.^26,40,42,43^ These assessments echo the viewpoint of a recent historian of 20th-century psychedelic research, who have argued that the scientific evaluation of research results in the field was meaningfully affected by worsening public sentiment, rather than by scientific quality or clinical utility.^5^

Classical psychedelic drugs are considered to act psychologically as meaning–response magnifiers^44,45^ or amplifiers of consciousness^46^ that increase the psychological intensity of experience. Accordingly, the context of their use is probably a key factor in any sustained psychological effects, whether positive or negative.^47^ Like most drugs in medical usage, they may be helpful when used in tailored settings by certain individuals but harmful in other circumstances.

Thus, individual anecdotes characterising the results of psychedelic use should be weighted accordingly, because these may suggest unrealistic projections of the results of psychedelic drugs being used at scale within specific circumscribed settings. However, dramatic individual cases – regardless of whether the outcome is positive or negative – often attract attention in the popular media.^2,18,26,37,48–51^ Media outlets are directly incentivised to maximise attention, probably to an even greater degree in the social media era. This incentive structure can contribute to a biased dissemination of information, which may contribute to excessive hype or moral panic regarding psychedelic drugs.^2,19^ In this context, it is especially crucial for progress within the field of psychedelic medicine that researchers, clinicians, regulators and policy-makers focus on aggregated scientific evidence as the primary source of information regarding the potential therapeutic applications of psychedelic drugs.

## Supporting information

Bender et al. supplementary material Bender et al. supplementary material

## Data Availability

Data access statement: D.A.B. had full access to all the data in the study and takes responsibility for their integrity and the accuracy of the data analysis. Data-sharing statement: data will be shared upon request for research purposes at the discretion of the study authors.

## Supplementary material

The supplementary material is available online at https://doi.org/10.1192/bjo.2025.10974
