# Supplementary material for: Trends in the psychedelic renaissance: applying artificial intelligence to measure media portrayal of psychedelic drugs in the 21st century
Source: BJPsych Open. 2026 Feb 12;12(2):e63. doi: 10.1192/bjo.2025.10974 (PMC12912910; doi:10.1192/bjo.2025.10974)
Supplement: Bender et al. supplementary material [file S2056472425109745sup001.pdf]

Supplementary Methods

ChatGPT Analysis

After inserting the prompt "Please tell me whether the article below primarily pertains to the therapeutic potential of psychedelic drugs.", the article content was provided to the AI. On the basis of AI response, Yes or No was coded for the individual URL by human raters. If the AI response was considered ambiguous by the human rater, this procedure was repeated up to two additional times until the AI produced a clear response. If a clear response was not produced after 3 iterations, the AI was prompted, "Please tell me whether the article below primarily pertains to the therapeutic potential of psychedelic drugs. Please answer in one word, Yes or No.", and the response to this question was recorded. Human raters also recorded the date, title, and publication for each article.

For URLs judged by the AI to focus on the therapeutic potential of psychedelic drugs, the prompt "On a scale of 1-100, how positive is the article below about the therapeutic potential of psychedelic drugs, with 100=very positive and 1=very negative?" was provided to the AI, and the article title and text were then inserted. In response, the AI provided a numerical score which was recorded for each article. If the output included a score range instead of a specific score, the number at the lowest end of the range was recorded by convention.

Total URL Determination

To determine the total number of URLs which appear for the search term "psychedelics" on news.google.com for a given year, the total number of URLs appearing in the search (not accounting for duplicates) was recorded. If 300 URLs appeared for a search that was confined to a given calendar year (the maximum URLs that Google presented for a given search), searches were confined to shorter temporal periods until each individual search produced under 300 URLs. URLs for each shorter period encompassing the year in its entirety were then summed to determine the total number of URLs for a given calendar year.

Top 50 English Language Media Website Determination

The top 50 most trafficked English-language media websites were sourced from pressgazette.co.uk using the month February 2025 (Supplementary Materials, Appendix F).

Supplementary Materials

Appendix A: Excluded URL Categories

Reason for Exclusion	Total URLs Excluded
Scientific journal article	183
Insufficient text	118
Duplicate of included article	68
No publication date	44
Excessive/insurmountable paywall	14
Nonfunctional URL	8
Not published during year of search	2
Advertisement	2

Scientific journal articles were published in peer-reviewed journals, and thus were not considered to be general media articles. Media articles describing the results of scientific publications for a general audience were included. Excessive/insurmountable paywalls had high costs to see an article, or it was not possible to circumvent the paywall without unique credentials. Nonfunctional URLs appeared on the Google search but it was impossible to access the URL offered by the search engine.

Appendix B: Human Rater Article Subgroup Information

Subgroup 1

a. Human Raters:
   1. Juy Yusuf
   2. Suraj Shankar
   3. Syed Ali Ahmed
   4. Brandon Kiley
   5. Amanda Pekau
   6. Jacob Steinle
   7. Medha Iyer
   8. Sara Martin
   9. Nithya Pippala

b. List of Articles Rated (Sentiment Scores):

https://www.utne.com/mind-and-body/harm-reduction-zm0z14uzlin/

https://www.fool.com/investing/2023/09/11/pharma-psychedelics-stock-dont-buy-otsuka/

https://www.vice.com/en/article/its-time-to-start-studying-the-downside-of-psychedelics/

https://www.philanthropy.com/article/a-psychedelic-renaissance/

https://www.cbc.ca/news/canada/newfoundland-labrador/hallucinogen-being-offered-by-st-john-s-man-to-treat-addiction-1.2706786

https://www.msn.com/en-us/health/other/psychedelic-therapy-from-taboo-to-mainstream-healthcare/ar-BB1rnSXH

https://www.usatoday.com/story/life/health-wellness/2021/07/26/megan-fox-christina-haack-tried-ayahuasca-psychedelics-what-know/7959203002/?gnt-cfr=1&gca-cat=p

https://www.theatlantic.com/ideas/archive/2024/10/psychedelics-medicine-science/680286/

https://www.ft.com/content/4da607e4-c2af-4002-ab96-cfe79269aff2

https://www.mic.com/life/can-microdosing-psychedelics-take-the-edge-off-a-first-date-18794357

https://web.archive.org/web/20250215005927/https://eu.detroitnews.com/story/news/local/detroit-city/2025/02/09/inside-legal-battle-rages-detroit-and-psychedelic-mushroom-using-church/77978805007/

https://theconversation.com/how-lsd-helped-us-probe-what-the-sense-of-self-looks-like-in-the-brain-57703

https://www.wordonfire.org/articles/psychedelics-antinatalism-and-dostoevsky/

https://www.citizen.org/article/weak-evidence-for-psychedelic-drugs-as-potential-treatments-for-depression-and-other-mental-illnesses/

https://www.businessinsider.com/list-top-psychedelics-companies-by-funding-2021

https://www.sciencenordic.com/brain-denmark-future-health/psychedelic-mushrooms-effect-on-the-brain-to-be-tested-in-new-study/1421592

https://www.teenvogue.com/story/people-with-depression-and-anxiety-could-benefit-from-hallucinogens

https://www.nytimes.com/2024/08/23/opinion/psychedelics-mdma-mental-health.html

https://sfstandard.com/2024/11/06/psychedelic-major-degree-undergraduate-ciis/

https://www.augustachronicle.com/story/news/nation-world/2008/03/12/nat-190750-shtml/64546256007/

https://www.bostonmagazine.com/2020/02/06/legalize-magic-mushrooms-massachusetts/

https://www.theguardian.com/world/2008/mar/05/religion.israelandthepalestinians

https://www.cbc.ca/news/canada/british-columbia/bc-sexual-abuse-psychedelic-therapy-1.5953480

https://www.vice.com/en/article/the-indigenous-mexican-tribe-that-honors-rare-psychedelic-toads/

ICC (2,k), Human: 0.56
ICC (2,k) AI: 0.94

c. List of Articles Rated (Whether Article Focuses on the Therapeutic Potential of Psychedelic Drugs):

https://maps.org/news/bulletin/psychedelics-and-parentingfinding-connection-in-a-disconnected-world/

https://journalistsresource.org/home/psychedelics-research-roundup/

https://www.washingtonpost.com/made-by-history/2023/06/29/psychedelics-drugs/

https://theconversation.com/psychedelics-researchers-balance-trippyness-with-scientific-rigor-after-history-of-legal-and-cultural-controversy-podcast-191502

https://www.politico.com/newsletters/future-pulse/2024/11/11/the-buzz-about-trump-and-psychedelics-00188669

https://www.statesman.com/story/news/2016/09/23/police-seize-large-stash-of-psychedelic-mushrooms-in-south-austin-home/9907112007/

https://www.business-standard.com/article/news-ians/dreaming-like-being-on-a-drug-trip-study-114070300719_1.html

https://www.udiscovermusic.com/stories/the-bosstown-sound/

https://psychedelicalpha.com/news/interview-with-shlomi-raz-ceo-and-chairman-of-eleusis

https://scitechdaily.com/psychedelic-breakthrough-scientists-successfully-achieve-total-synthesis-of-ibogaine/

Subgroup 2

a. Human Raters:
   1. Rana Abdalla
   2. Reetwan Bandyopadhyay
   3. Madeline Villaba
   4. Sophia Chertock
   5. Jatin Sridhar
   6. Harry Dunn
   7. Rishi Gorguntula
   8. Umer Jalil
   9. Baris Ercal
   10. Brendan Ross

b. List of Articles Rated (Sentiment Scores):

https://law.yale.edu/yls-today/news/panel-discusses-coalition-building-unlock-therapeutic-effects-psychedelics
https://www.njspotlightnews.org/2022/12/psilocybin-psychedelic-mushrooms-mdma-depression-anxiety-alcohol-use-suicide-cory-booker-oregon/
https://www.theguardian.com/commentisfree/2022/oct/18/psychedelic-drugs-us-republican-thiel-mercer-foundation
https://www.thecrimson.com/article/2024/10/22/mass-question-4-psychedelics/
https://www.businessinsider.com/psychedelics-trip-therapy-2018-1
https://www.pnas.org/post/journal-club/study-isolates-neurons-involved-anti-anxiety-but-not-hallucinogenic-effects-psychedelics
https://www.europeanpharmaceuticalreview.com/news/144774/positive-effects-of-psychedelic-microdosing-likely-due-to-placebo-effect-finds-study/
https://www.opendemocracy.net/en/transformation/political-significance-of-lsd/
https://www.politico.eu/article/navigate-high-low-psychedelic-therapy-lsd/
https://www.madinamerica.com/2021/09/psychedelics-new-psychiatric-craze/
https://www.nytimes.com/2025/02/04/health/fda-mdma-psychedelic-therapy-psymposia.html
https://www.cbc.ca/news/health/ayahuasca-faq-1.4631656
https://interestingengineering.com/science/7-fascinating-psychedelics-that-are-making-a-resurgence-in-scientific-research
https://www.vice.com/en/article/ego-death-is-the-trip-competitive-psychedelic-users-are-chasing/
https://apnews.com/article/mdma-psychedelic-drug-fda-ptsd-lykos-709b23613031fb11d3dc5228c8476a39
https://www.psypost.org/awe-inspiring-psychedelic-trips-reduce-narcissism-by-boosting-connectedness-and-empathy-study-suggests/
https://www.wired.com/story/magic-mushrooms-uk-psilocybin-cubensis-depression-treatment-prescription-psychedelic-drugs/
https://www.vice.com/en/article/hunting-for-the-most-potent-psychedelic-toad-venom-on-earth/
https://www.bps.org.uk/psychologist/looking-back-brief-history-psychedelic-psychiatry
https://www.rollingstone.com/culture/culture-news/psilocybin-legal-therapy-mdma-753946/
https://theconversation.com/psychedelics-could-make-mental-health-worse-in-people-with-a-personality-disorder-226099
https://vtdigger.org/2024/11/18/study-group-hesitant-about-psychedelic-therapy-in-vermont/
https://www.newscientist.com/article/2394987-psychedelic-health-claims-may-be-promoting-risky-self-medication/

ICC (2,k), Human: 0.50
ICC (2,k), AI: 0.93

c. List of Articles Rated (Whether the Article Focuses on the Therapeutic Potential of Psychedelic Drugs):

https://boltsmag.org/drug-referendums-november-2022/
https://www.npr.org/2023/11/05/1210327976/psychedelic-drugs-psilocybin-roland-griffiths-depression-cancer-meditation
https://www.independent.co.uk/news/science/scientists-dolphins-lsd-communitcate-with-them-experiment-talk-john-lilly-margaret-howe-lovatt-a7787556.html
https://news.harvard.edu/gazette/story/2024/05/study-of-psychedelics-announces-funding-recipients/
https://www.npr.org/sections/13.7/2014/10/02/352924426/can-psychedelics-expand-our-consciousness
https://www.rollingstone.com/music/music-news/arctic-monkeys-desert-journey-the-making-of-humbug-69144/
https://www.vice.com/en/article/a-very-psychedelic-conversation-with-the-creators-of-rick-and-morty/
https://www.asianscientist.com/2011/12/topnews/wwf-wild-mekong-report-new-species-elvis-monkey-self-cloning-female-lizard-psychedelic-gecko-2011/
https://www.technologynetworks.com/neuroscience/articles/to-hallucinate-or-not-the-big-questions-on-psychedelics-at-fens-2022-365377
https://www.washingtonpost.com/archive/local/2001/10/04/john-c-lilly/594dc878-43ac-43ae-a470-e17641d5b580/

Subgroup 3

a. Human Raters:
   1. Adeyemi Sola
   2. Arushi Mohite
   3. Melinda Daniels-Tineo
   4. Anvi Divekar
   5. Hannah Wang
   6. Sonali Sharma
   7. Madison Stille
   8. Gibson Werner
   9. Akila Anandarajah
   10. Mia Stonov

b. List of Articles Rated (Sentiment Scores):

https://www.marijuanamoment.net/virginia-house-panel-kills-psychedelics-bill-that-had-already-been-approved-by-the-senate/
https://www.dazeddigital.com/artsandculture/article/35803/1/talking-to-a-warlock-about-the-spiritual-power-of-ket
https://www.statnews.com/2023/07/03/psychedelics-ketamine-mdma-ptsd-how-they-work/
https://www.theguardian.com/us-news/2023/oct/07/california-governor-vetoes-bill-to-decriminalize-natural-psychedelic-drugs

https://www.forbes.com/sites/amandasiebert/2020/11/13/microdosing-psychedelics-is-trendy-but-does-it-work-heres-what-science-says/
https://harris-sliwoski.com/cannalawblog/how-religious-groups-can-petition-to-use-psychedelics-legally/
https://www.thedailybeast.com/psychedelic-hype-is-hurting-more-people-than-we-realize/
https://www.theguardian.com/science/2025/jan/11/white-people-shouldnt-mess-with-it-native-american-church-laments-psychedelic-cactus-shortage
https://www.golfdigest.com/story/why-serious-players-are-doing-psychedelics-like-mushrooms-and-ayahuasca
https://www.politico.com/news/2024/06/04/psychedelic-medicine-ptsd-fda-00161611
https://www.nytimes.com/2021/05/09/health/psychedelics-mdma-psilocybin-molly-mental-health.html
https://www.imperial.ac.uk/news/192046/science-microdosing-psychedelics-remains-patchy-anecdotal/
https://www.psychologytoday.com/intl/blog/stuck/201001/the-acid-wars
https://newsroom.clevelandclinic.org/2024/03/22/why-researchers-are-studying-use-of-psychedelics
https://www.pbs.org/newshour/health/psychedelic-drug-mdma-faces-questions-as-fda-considers-approval-for-ptsd
https://www.sciencealert.com/researchers-rank-recreational-drugs-based-on-how-dangerous-they-are
https://www.dailymail.co.uk/health/article-5631807/Inside-Mexican-psychedelic-drug-rehabs-scores-Americans-opioids.html
https://penntoday.upenn.edu/news/could-psychedelics-simultaneously-treat-chronic-pain-and-depression
https://www.nytimes.com/2022/08/23/health/marijuana-psychedelics-young-adults.html
https://psychiatryonline.org/doi/10.1176/appi.pn.2021.12.14
https://www.theguardian.com/world/2010/nov/23/godfather-ecstasy-alexander-shulgin-stroke
https://www.washingtonpost.com/business/2024/06/08/psychedelic-drugs-mdma-fda-therapy/
https://medicalxpress.com/news/2020-02-magic-mushrooms-lsd-depression-anxiety.html

ICC (2,k), Human: 0.51
ICC (2,k), AI: 0.93

c. List of Articles Rated (Whether the Article Focuses on the Therapeutic Potential of Psychedelic Drugs):

https://www.cbc.ca/radio/thecurrent/the-current-for-april-24-2018-1.4631421/dreams-visions-and-diarrhea-what-to-expect-if-you-take-ayahuasca-1.4632719
https://www.vice.com/en/article/life-is-a-cosmic-giggle-803-v18n5/
https://maps.org/news/bulletin/clemency-for-deadheads-and-others-in-prison-for-non-violent-drug-offenses/
https://www.theguardian.com/science/2012/jun/28/psychedelic-drugs-mysteries-brain-government-adviser
https://www.axios.com/2021/04/26/psychedelic-drug-developer-mindmed-public

https://www.independent.com/2009/04/24/dalai-lama-imparts-wisdom-ucsb-crowd/
https://www.wbur.org/news/2024/09/20/massachusetts-election-psychedelic-mushrooms-ballot-question-4-explainer

https://reason.com/2015/11/06/friday-av-club-you-are-watching-lsd-tv/

https://www.statnews.com/2020/01/07/transforming-psychedelics-into-mainstream-medicines/
https://www.proactiveinvestors.com/companies/news/1065842/atai-life-sciences-backed-psychedelic-drug-could-offer-superior-value-analysts-believe-1065842.html

Appendix C: Instructions to Human Raters for Sentiment Rating

Identical instructions as given to the AI for sentiment scoring were sent via email to all human raters. The specific content of the email is included below.

“Sentiment analysis: This will be for 24 articles on the attached Excel document…read these articles and for each article answer the question, "On a scale of 1-100, how positive is the article below about the therapeutic potential of psychedelic drugs, with 100=very positive and 1=very negative?" I can't really give any more guidance on how to do this other than: do your best, there's no "right answer," just give your honest opinion—and make sure to give a number no matter what, even if you're not sure!”

Appendix D: Total Articles Indexed for the Search Term “psychedelics” on Harvard Media Cloud and Google News, 2014-2025

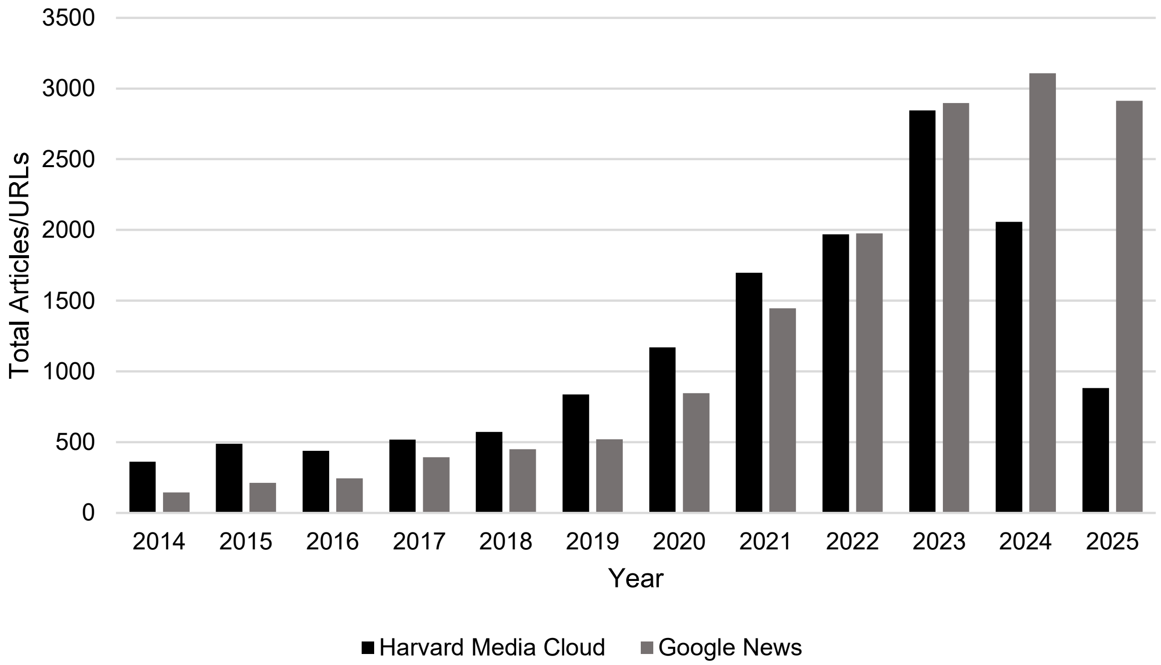

Total URLs from Google News were sourced from each calendar year per *Methods.* Articles from Harvard Media Cloud were sourced from https://search.mediacloud.org/ using the search term “psychedelics” for individual calendar years from 2014-2025 for the combined United States-National and United Kingdom-National datasets. For the year 2025, total articles/URLs were determined for both databases by multiplying the total from 1/2025-3/2025 by 4. Articles prior to 2014 were not indexed by Harvard Media Cloud.

Appendix E: All Represented Publications

5280
032c
10 Zen Monkeys
24 News
303 Magazine
34th Street
6sqft New York City
7 News WWNYTV
8 News Now
A Way To Garden
ABC 11
ABC 12 News
ABC 33/40 News
ABC 6 On Your Side
ABC 7 New York
ABC News
ABC News Australia
ABC30 Fresno
ABC7
Access Newswire
AddictionCenter
Additude
Aeon
afaqs!
Aidsmap
AJC
AJMC
Al Jazeera
AL.com
Alabama Local News
Alabama Political Reporter
Alaska Public Media
Alcalde
Aljazeera
All Thats Interesting
allure
American Association For The Advancement of Scier
American Legion
American Psychological Association
American Society for Microbiology
American University
Amplitude
Anacortes American
Analog Planet
Ancient Origin
Cascade PBS
Cato Institute
CBC
CBS News
CBS8
CDM
Cedars Sinai
Celeb Stoner
Centre Daily Times
Charleston City Paper
Chemical & Engineering News
Chemistry World
Chicago Magazine
Chicago Reader
Chicago Tribune
Christianity Today
Chron
Cision
City Pulse
Cleveland Clinic
Cleveland.com
Climbing
Clinical Advisor
Clinical Leader
Clinical Trials Arena
CNBC
CNET
CNN
Coconuts Jakarta
Colorado Newsline
Colorado Public Radio
Coloradoan
Colossal
Columbia Magazine
Columbia News
Columbia University Department of Psychiatry
Columbus Dispatch
Common Wealth Beacon
Communications Psychology
Complex
Conde Nast Traveler
Cool Hunting
Cornell Chronicle
CorvetteBlogger
Cosmos Magazine

Angry Metal Guy
AnOther Magazine
Anschutz
AP News
Arch Daily
Architectural Digest
Architecture, Au
Arizona Daily Star: Tuscon
Army Times
arstechnica
Artnet
ARTNews
Artsy
ASCO Daily News
Asian Scientist
Atlanta Jewish Times
Atlas Obscura
Austin American Statesman
Austin Monitor
Austin Monthly
Australian Broadcasting Corporation
Australian Geographic
Auto Evolution
AV Club
Axios
Axios Boston
Axios Denver
Backstage
Bandcamp
Bay Area News Group
Bay Nature Magazine
BBC
Behavioral Scientist
Benzinga
Berkeley Library
Berkeley Public Health
Berkeleyside
Best
Big Think
Billy Penn at WHYY
Binghamton University (BingU News)
BioMed Central
BioPharma Dive
BioSpace
BioTechniques
COUNTERPUNCH
Courthouse News Service
CPR News
Creative Boom
CryptoPsychedelic
CT Insider
CTV News
CU Denver News
Cult MTL
D Magazine
Daily Bruin
Daily Bulldog
Daily Dot
Daily Mail
Daily Nexus
Dallas News
Dallas Observer
Dance Music NW
Dancing Astronaut
Dangerous Minds
DAV
Dazed
DCist
DEA
Delano
Delish
Democrat & Chronicle
Dentons
Denver 7
Deseret News
Desert Publications: Palm Springs Life
Desert Sun
Design Boom
Destructoid
Detroit Eater
Detroit Free Press
Devon Live
Dezeen
Diffuser
Digiday
Disabled American Veterans
Discover Magazine
Document Journal
Dorchester Reporter
drownedinsound

Bitcoin.com News
Block Club Chicago
Bloody Elbow
Bloomberg
BMC Medical Education
BMJ Group
Boing Boing
Bolts
Boston Globe Media Partners
Boston Herald
Boston Magazine
Boston News
Boston University
Boston.com
Boulder Weekly
Box Office Prophets
BPS
Brain & Behavior Research Foundation
BreakingAC.com
British Journal of Sports Medecine
Brooklyn Vegan
Bulletin of the Atomic Scientists
Burlington Free Press

Business Insider
Business Insider India
Business Standard
Business Wire
BuzzFeed
c&en
Cal Alumni Association
Cal Matters
camh
Cape Cod Times
Car Buzz
Cardiology Today
Cartoon Brew

Drug Discovery & Development
Drug Science
Drug Target Review
Duke University School of Medicine
DW Health
E News
Earth.com
edm.com
Edmonton CityNews
Edmonton Journal
eJewish Philanthropy
El País
Electronic Beats
Emory University
Endpoints News
Entertainment Weekly
Entrepreneur
ERR
Esquire
Essence
ET
ETF.com
EurekAlert!

Eurogamer
European Pharmaceutical Review
Exclaim!
Express
Fast Company
Fatherly
Festival Insights
Fierce Biotech
Fierce Pharma
Filter
Financial Review
FindLaw
Firehouse

Firstpost
Flavor Wire
Florida Politics
Flyer News
Foley and Lardner LLP
Folha de S.Paulo
Forbes
Fortune
Forward
Foster's Daily Democrat
Fox 5 Washington DC
Fox 9 KMSP
Fox 9 Minneapolis
Fox News
Fox San Antonio
Fox Sports
FOX9 KMSP
Freethink
Fresh Water Cleveland
Futurism
Futurity
Ganjapreneur
GeekWire
GeneOnline
Genetic Engineering and Biotechnology News
Give to UC Davis
Glasstire
Global News
Global Newswire
Globan Voices
Golf Digest
Good Times
Governing
GQ
Grand Fork Herald
Grantland
GratefulWeb
Gray Local Media Station
Greater Good Science Center
Greenville News
Grist
H.S. Gere & Sons, Inc.
Hagerty
Hammer And Rails
Harris and Sliwoski
KQED News (NPR)
KSL.com
KSNV
KTLA
KVAL
KWTX
kxan
LA Review of Books
LABIOTECH
Labroots
LAist
Lancaster Online
Las Vegas Optic
Las Vegas Weekly
Laughing Squid
Law.com
Liberal Currents
Life Science Leader
Listverse
Little Black Book
Littler
Live For Live Music
Livescience
London Real
London School of Economics
Londonist
LoneStar Live
Longreads
Los Angeles Magazine
Los Angeles Review of Books
Louder
Louder than War
Loudwire
Lucid News
Mad in America
Maddyness
Magic Wands: HaydenShapes' Psychedelic Germ
Mail&Guardian
Maple Ridge-Pitt Meadows News
Marie Claire
Marijuana Moment
MarketWatch
Mashable
Mass General
Mass Live

Hartford Health Care
Harvard Business Review
Harvard Health Publishing
Harvard Law Today
HCP Live
Healio
Healthcare Brew
Healthline
Hearst Newspapers Inc.
High Times
Holland and Knight
Hone
Hopkins Brainwise
Hospice News
HOT ROD Network
Houston Chronicle
Houston Public Media
Houstonian Magazine
HS Insider
HUB
Huck Magazine
HuffPost
Husch Blackwell
Hypebeast
Hyperallergic
iai news
ICEERS
ICON
ICT News
IFLScience
IGN
iHeart
Imperial College London
Inc
Indie102.3
IndieWire
Indy Week
InMenlo
Inside Hook
Insider Monkey
Interesting Engineering
International Business Times
Interview Magazine
Inverse
Iowa Public Radio
Massachusetts General Hospital
Massive Science
McGill University
Med Central
Med Shadow Foundation
Medical Humanities Blog
Medical Press
Medical University of South Carolina
Medical Xpress
MedicalNewsToday
Medium
MedPage Today
Medscape
Men's Fitness
Men's Health
Men's Journal
Mental Health Today
Metro
Mg Magazine
Miami Herald
MIC
Michigan Live
Michigan Medicine
Michigan State University
MicroDose
MIT Sloan Management Review
MIT Technology Review
MixMag
MJBizDaily
MJH Life Sciences
mobi health news
Modern Drummer
Moffly Media
Monash University
Mondaq
Monochrome
Monster Children
Montana Free Pass
Monterey County NOW
Monthly Portland
Morning Brew
Mother Jones
Motorcycle & Powersports News
MotorTrend
Mount Sinai

ISRAEL21c
Israel365 News
It's Nice That
Jacobin
Jambands
Jewish Telegraphic Agency
Johns Hopkins Magazine
Johns Hopkins Medicine
Johns Hopkins University Hub
Jstor Daily
Jurist News
Just Jared
jweekly
K5 Seattle
KALW
KATU ABC 2 News
KCRA
KCRW
KDVR
KEYE
KFF Health News
KFOX14 News
KGET

KGW
KHQ
King 5
King's College London
KITV
KJZZ
KMVT
KNKX
knowable Magazine
KOAA News 5
KOAT
KOMO News
KPBS

MPR News
MSN
Muckrock
Mugglehead Magazine
Multidisciplinary Association for Psychedelic Studies
MUSC News
My Modern Met
My San Antonio
NASA
National Audubon Society
National Geographic
National Review
Nature News
Nautilus
NBC 5 Chicago
NBC 5 Dallas-Fort Worth
NBC Boston
NBC Connecticut
NBC Montana
NBC New York
NBC News
NBC10 Boston
NBC15 News

Neoscope
NetworkWorld
Neuroscience News
Nevada Current
New Internationalist
New Statesman
New York Magazine
Newcity Music
Newport Academy
News @ The U
News Atlas
News Medical
News Nation

news24
NewScientist
NewsGP
Newsweek
NexStar Media
Next Avenue
Next Pittsburgh
NIH
Nintendo Life
NJ Spotlight News
NJ.com
NME
No Echo
North Eastern Global News
NorthCentral PA News
northjersey.com
Northwest Arkansas Democrat Gazette
Northwest Public Broadcasting
NPR
NPR Michigan
NPR Nevada
NPR Texas
NYU
NYU Langone News
NZ Herald
Observer
Ohio State News
OkayAfrica
ON Sports Illustrated
Open Access Government
Open Culture
Open Democracy
OpenPR
Oregon Capital Chronicle
Oregon Live
Oregon News
Oregon Public Broadcasting
Orion
Out Magazine
Outside Magazine
Ozarks First News
Pacific Standard
Page Six
Palo Alto Online
Parkinsons News Today
Sci Tech Daily
Science
Science Alert
Science Business
Science Focus
Science Friday
Science News
ScienceBlogs
ScienceNordic
Scientific American
SciTechDaily
Screenrant
sd metro
Seattle Weekly
Seeking Alpha
Semafor
Sentinel and Enterprise
SF Weekly
SFGate
Sifted
Silent Radio
Sky News
Slant Magazine
Slate
Slicing Up Eyeballs
Slug Mag
Smith College
Smithsonian Magazine
Sneaker Bar Detroit
Snopes
Soap Central
Sociedelic
Sojourners
Sonic Perspectives
Soundsandcolours
South China Morning Post
SouthCoastToday
Southern California Public Radio
SouthernMinn.com
Space.com
Spectrum News
Spectrum News NY1
Spin
Sports Illustrated
Stanford Medicine News Center

Paste Magazine
Patch
Patheos
PBS News
PBS SoCal
Penguin Random House
Penn Medicine News
Penn Program on Regulation
Penn Today
PetaPixel
Pharma Phorum
Pharma Voice
Pharmaceutical Technology
Pharmacy Times
Philly Voice
Phys.org
Piedmont Exedra
Pinkbike
Pitchfork
Plaid Zebra
PNAS Journal Club
Political Critique
Politico
Polygon
PopMatters
Popular Mechanics
Popular Science
Portland Press Herald
Post Independent
Poughkeepsie Journal
Print Magazine
Proactive Group Holdings
Project CBD
Prospect
Proto Life
Psych Central
Psychedelic Alpha
Psychiatric News
Psychiatric Times
Psychiatrist.com
Psychiatry Advisor
Psychiatry Online
Psychology Today
PsyPost
Public Citizen
Stanford University
Star Tribune
Stars and Stripes
STAT News
Steamboat Pilot and Today
Stereogum
stlpr
StockHouse
Straight Arrow News
straight.com
Stuff NZ
Summit Daily
swissinfo.ch
SYFY
Synbiobeta
Tacoma News Tribune
Tacoma Weekly
Taipei Times
Talking Drugs
TCNJ Signal News
Technology Networks
TechTarget
TecScience
TED
Teen Vogue
Telegram & Gazette
Texas Monthly
Texas Observer
Texas Standard
The Alliance Review
The American Conservative
The American Journal of Managed Care
The Architect's Newspaper
The Arts Desk
The Arts Fuse
The Aspen Times
The Associated Press
The Atlanta Journal-Constitution
The Atlantic
The Augusta Chronicle
The Austin Chronicle
The Badger Herald
The Boston Globe
The Bottom Line UCSB
The Bowdoin Orient

Purdue Exponent
Purdue University
Quartz
Quillette
RA
Raconteur
Reactor Magazine
Reason
Red Bull
ReedSmith
Refinery29
Regents of the University of Colorado
Regina Lead-Post
Regulatory Oversight
Relix
Reno Gazettte Jounral
Reuters
Reveal News
Reverse Shot
RFI
Riverfront Times
Road to VR
Roads & Kingdoms
Robb Report

Rockstar Games
Rolling Stone
Royal Australian College of General Practitioners
RVA
Salon
San Francisco Chronicle
Santa Cruz Sentinel
Saskatoon Star Phoenix
Savannah Now: Savannah Morning News
SBNation
SBS News Australia

The British Psychological Society
The Brown Daily Herald
The Bulletin
The California Aggie
The Catalyst News
The Center Square
The Chicago Reporter
The Christian Post
The Chronicle of Philanthropy
The Colorado Sun
The Conversation
The Criterion Collection
The Cut
The Daily Beacon: The University of Tennesee
The Daily Beast
The Daily Evergreen
The Dallas Morning News
The Denver Post
The Detroit News
The Duke Today
The Durango Herald
The Economist
The Elm

The Exponent
The Face
The Film Magazine
The Financial Times
The Fresno Bee
The Gazette
The Georgia Straight
The Globe
The Globe and Mail
The Growth Op
The Guardian
The Harvard Crimson

The Harvard Gazette
The Harvard Magazine
The Hill
The Hindu
The Hollywood Reporter
The Independent
The Indian Express
The Inquiry
The Insurance Business Magazine
The Intercept
The Irish Independent
The Irish Times
The Jerusalem Post
The Johns Hopkins Newsletter
The Jordan Times
The Journalist's Resource
The Kellogg Insight
The Line of Best Fit
The Link Newspaper
The Litter
The Los Angeles Times
The Ludlow Cub
The Mandarin
The Mary Sue
The Mercury News
The Military Times
The Mirror
The Morning Journal
The Morning News
The Motley Fool
The Nation
The National Law Review
The Nevada Independent
The New Bedford Light
The New Indian Express
The New Republic
The New York Post
The New York Times
The New Yorker
The News & Observer
The Next Web
The Olympian
The OPB
The Orange County Register
The Oregonian
UC Davis
UC News
UC San Diego Magazine
UC San Diego Today
UC Santa Cruz
UCF Today
UCHealth Today
UChicago News
UCR
UCSF
UDiscover Music
UK Parliament Post
Ultimate Classic Rock
UMKC
UNC Health News
Uncut
Undark
UnHerd
United Nations Office on Drugs and Crime
Univeristy Affairs
Universe Today
University College London
University of Alberta
University of Calgary
University of California
University of Cincinati News
University of Colorado Anschutz Medical Campus
University of Colorado Boulder
University of Colorado Cancer Center
University of Maryland School of Medicine
University of Miami Miller School of Medicine
University of Michigan
University of Nebraska Medical Center
University of Oregon - OregonNews
University of Saskatchewan
University of South Florida
University of Texas Dell Medical School
University of Toronto News
University of Utah Health
University of Wollongong News
UNM Health Sciences Newsroom
US News
USA Today
USask
USF Health

The Palm Beach Post
The Pennsylvania Capital-Star
The Pharmaceutical Journal
The Press Democrat
The Progressive
The Provincetown Independent
The Quanta Magazine
The Quietus
The Register
The Register-Guard
The Regulatory Review
The Sacramento Bee
The San Francisco Standard
The Santa Barbara Independent
The Scientist
The Shorthorn
The Signal
The Siskiyou
The Sonoma Index-Tribune
The Source
The Spaces
The Spokesman-Review
The Standard
The Stranger
The Strategist
The Swaddle
The Sydney Morning Herald
The Telegraph
The Texas Tribune
The Times
The Times of Israel
The Tribune
The University News
The University of Alabama at Birmingham
The University of British Columbia
The University of Queensland
The University of Western Australia
The Varsity
The Verge
The Vintage News
The Vinyl Factory
The Virgin
The Wall Street Journal
The War Horse News, Inc
The Wash
UTNE
UToronto
Vancouver Sun
Vanderbilt University Law School
Vanity Fair
Variety
VCU News
Venture Beat
Vermont Public
Very Well Mind
Verywell Health
VG247
VICE
Virginia Mercury
Virginia Tech News
Visual Capitalist
VMI
Vogue
Voice of America News
Volteface
Volume One
Vox
Vox Media: Blazer's Edge
VPM News
Vtdigger
WABE
WAMU 88.5 American University Radio
Washingtonian
WashU Medicine
WashU The Source
WBRC News
wbur
WCAX3
WCVB Boston
WDBJ7
WDTV
Wealth Professional
Weill Cornell Medicine Newsroom
Well+Good
Westside Seattle
WestWord
WFTV
WFTV9
WGBH
WhaTech

The Washington Post
The Weather Channel
The Week
The Wrap
thebmjopinion
thereview
TheScientist
THINK (NBC)
Thred
Thrillist
TIDAL
TIME
Times Higher Education
Times of Malta
TMC News
Toiletovhell
Toronto Life
Toronto Star
Town and Country
TownLift Park City
Travel Weekly
Treatment Magazine
Treble

TrendHunter
Tricycle: The Buddhist Review
truthdig
TTAC
Tulane University
Tuscaloosa News
U.S. Customs and Border Protection
U.S. News & World Report
UAB News
UBC Okanagan News
UC Berkeley News
UC Berkley Berkleyside

Whoop
WHYY News (PBS/NPR)
Willamette Week
Wired
Wisconsin Public Radio
Wiwibloggs
WLRN (NPR)
WLWT5
WNWO
Women's Wear Daily
Word on Fire
WOSU Public Media (NPR)
WPDH
WRGB
WTMJTV- Milwaukee
WTOL 11
wttw
WUNC North Carolina Public Radio
WUR
WUSF NPR
WVTM13
WWE
WYFF4

Wyoming Public Media
Yahoo Life UK
Yahoo! Finance
Yahoo! News
Yale Law School
Yale News
Yale School of Medicine
YNet News
Yo! Venice!
Yoga Journal
YouGov
ZME Science

Appendix F: Publications in the Top 50 Most Trafficked English Language Media Websites Included Within the Database

1. ABC News
2. ABC News Australia
3. AP News
4. BBC
5. Business Insider
6. BuzzFeed
7. CBC
8. CBS News
9. CNBC
10. CNN
11. Daily Mail
12. Express
13. Forbes
14. Fortune
15. Fox News
16. HuffPost
17. MSN
18. NBC News
19. New York Post
20. Newsweek
21. Sky News
22. The Guardian
23. The Hill
24. The Hindu
25. The Independent
26. The Indian Express
27. The Mirror
28. The New York Times
29. The Telegraph
30. The Wall Street Journal
31. The Washington Post
32. USA Today
33. Yahoo! Finance
34. Yahoo! News

The top 50 publications were sourced from pressgazette.co.uk using the most accessed sites during February 2025.

Appendix G: Data for Individual Publications

Only publications with at least 5 publications in the database are included in the table. % TP refers to the percentage of all articles for a given publication that were judged by the AI as focusing on the therapeutic potential of psychedelics.

Publication	Total Articles	% TP	Avg. Sentiment	Min.	Max.	St. Dev.
Vice	118	53%	75.6	35	90	11.2
The Guardian	109	56%	71.6	30	90	12.0
The New York Times	74	65%	76.4	40	85	11.3
MAPS	68	81%	85.3	75	95	3.7
Business Insider	64	83%	79.9	60	85	6.5
Forbes	56	80%	79.8	35	85	10.3
Rolling Stone	52	42%	80.5	60	85	6.5
NPR	50	62%	77.3	40	90	10.8
BBC	44	52%	73.7	60	90	10.0
Wired	40	55%	76.4	55	85	9.0
Marijuana Moment	38	79%	80.3	40	85	8.8
PsyPost	37	81%	78.6	65	90	7.6
Psychology Today	35	89%	77.7	60	90	8.0
The Conversation	34	88%	77.5	55	85	12.2
Psychedelic Alpha	33	82%	78.3	55	85	9.6
The Independent	30	67%	80.8	70	90	6.7
CBC	30	83%	72.6	40	85	13.2
CBS News	27	85%	79.6	40	85	10.1
The Washington Post	26	65%	75.3	60	90	11.2
New Atlas	25	84%	81.0	70	90	6.6
NewScientist	25	64%	75.9	55	85	7.8
NBC News	23	61%	76.1	60	90	9.1
Scientific American	22	100%	78.4	60	90	7.9
Vox	22	82%	76.9	55	85	9.3
Nature News	22	100%	76.2	40	85	11.2
Pitchfork	21	0%	N/A	N/A	N/A	N/A
Technology Networks	20	100%	80.3	65	90	7.0
Bloomberg	20	70%	76.8	60	90	8.2
Livescience	19	68%	74.6	60	85	6.9
MedicalNewsToday	19	100%	74.5	50	85	9.4
Newsweek	19	63%	72.5	55	85	8.3
Johns Hopkins Medicine	18	100%	82.5	70	90	5.8
CNN	18	89%	80.9	65	90	6.4
Dangerous Minds	18	6%	30.0	30	30	0.0
The Denver Post	17	53%	72.8	60	85	9.7
Imperial College London	16	94%	80.3	35	85	12.5
Big Think	16	69%	79.6	65	85	7.6
Patch	16	44%	79.3	70	85	7.3
STAT News	15	73%	73.8	45	90	13.2
AP News	15	80%	72.5	40	85	13.2
The Atlantic	15	73%	72.3	35	90	15.9
Slate	14	43%	82.5	75	85	4.2
HuffPost	14	43%	80.0	75	85	4.5

wbur	14	71%	77.0	70	85	6.3
News Medical	14	86%	75.0	50	85	10.4
Daily Mail	14	36%	67.0	55	85	11.5
Inverse	13	85%	83.2	75	85	3.9
Medical Xpress	13	92%	81.7	70	90	5.9
Time	13	85%	79.1	60	95	11.4
PBS News	13	77%	77.5	40	85	14.2
The Financial Times	13	85%	75.0	55	85	10.0
Politico	13	85%	73.6	35	85	14.3
NME	12	0%	N/A	N/A	N/A	N/A
The Wall Street Journal	12	83%	76.5	60	85	9.1
Quartz	12	75%	75.6	55	85	10.1
The Los Angeles Times	12	67%	70.6	60	85	8.6
San Francisco Chronicle	11	91%	81.0	60	85	8.1
The Quietus	11	18%	80.0	80	80	0.0
Healthline	11	100%	79.1	65	85	8.6
The New Yorker	11	64%	78.6	50	90	13.5
The Harvard Crimson	11	64%	76.4	65	85	8.0
Wisconsin Public Radio	10	100%	83.5	60	90	8.5
UC Davis	10	90%	81.5	70	90	7.1
The Harvard Gazette	10	90%	81.1	60	85	8.6
Neuroscience News	10	100%	78.5	60	90	8.4
KQED News (NPR)	10	80%	76.9	70	85	5.6
Reason	10	50%	75.0	60	85	10.6
ABC News Australia	10	60%	73.3	60	85	8.2
UC Berkeley News	9	89%	85.6	85	90	1.8
BioSpace	9	89%	81.9	70	90	6.5
The Economist	9	78%	77.5	70	85	6.6
USA Today	9	56%	77.0	75	85	4.5
IFLScience	9	78%	75.7	70	80	3.2
Boing Boing	8	50%	86.3	75	95	8.5
GQ	8	50%	85.0	85	85	0.0
Dazed	8	38%	85.0	85	85	0.0
Hyperallergic	8	25%	85.0	85	85	0.0
SFGate	8	13%	85.0	85	85	N/A
Aeon	8	63%	80.0	70	90	9.4
Vogue	8	38%	80.0	70	85	8.7
Axios	8	100%	78.8	75	85	4.4
Fortune	8	75%	77.5	70	85	6.1
Yahoo! Finance	8	63%	74.0	50	85	16.0
The Daily Beast	8	50%	72.5	35	85	25.0
Salon	7	57%	83.8	75	90	6.3
The Regulatory Review	7	71%	81.0	75	85	4.8
Discover Magazine	7	57%	78.8	65	85	9.5
The Times of Israel	7	43%	78.3	65	85	11.5

Reuters	7	86%	78.3	75	85	5.2
Refinery29	7	71%	77.0	65	85	7.6
CPR News	7	100%	76.4	65	85	7.5
Smithsonian Magazine	7	29%	75.0	65	85	12.8
Science	7	86%	68.3	50	80	11.3
My Modern Met	6	0%	N/A	N/A	N/A	N/A
Healio	6	100%	80.8	70	85	6.7
Popular Science	6	67%	80.0	75	90	7.1
South China Morning Post	6	50%	80.0	75	85	5.0
British Psychological Society	6	100%	77.5	70	85	6.9
UTNE	6	33%	77.5	75	80	3.5
The Verge	6	50%	76.7	70	90	11.5
ABC News	6	50%	73.3	50	85	20.2
Medscape	6	100%	71.7	55	85	9.0
Bandcamp	5	0%	N/A	N/A	N/A	N/A
It's Nice That	5	0%	N/A	N/A	N/A	N/A
Laughing Squid	5	0%	N/A	N/A	N/A	N/A
Michigan Live	5	60%	85.0	85	85	0.0
Washingtonian	5	60%	85.0	85	85	0.0
Business Wire	5	100%	84.0	75	90	5.5
Johns Hopkins University Hub	5	100%	83.0	70	90	6.8
Observer	5	40%	82.5	80	85	2.5
Massive Science	5	100%	81.0	75	85	4.9
MPR News	5	100%	81.0	75	85	4.9
New York Post	5	60%	73.3	70	80	4.7

Appendix H: AI Sentiment Score Correlations with Human Raters

Rater	Article Subgroup	Education Level	Correlation Coefficient
Adebusola Adeyemi	3	Undergraduate	0.93
Baris Ercal	2	M.D.	0.87
Reetwan Bandyopadhyay	2	Undergraduate	0.86
Anvi Divekar	3	Undergraduate	0.85
Suraj Shankar	1	M.D.	0.84
Syed Ali Ahmed	1	Masters	0.84
Akila Anandarajah	3	Undergraduate	0.83
Gibson Werner	3	High School	0.82
Brendan Ross	2	M.D.	0.81
Arushi Mohite	3	High School	0.79
Brandon Kiley	1	M.D.	0.79
Sara Martin	1	M.D.	0.79
Medha Iyer	1	Masters	0.76
Harry Dunn	2	Undergraduate	0.74
Rishi Gorguntula	2	M.D.	0.72
Juy Yusuf	1	Undergraduate	0.7
Madeline Villaba	2	Undergraduate	0.69
Sophia Chertock	2	Undergraduate	0.69
Melinda Daniels-Tineo	3	Undergraduate	0.68
Jatin Sridhar	2	High School	0.68
Jacob Steinle	1	Medical Degree	0.65
Madison Stille	3	High School	0.55
Nithya Pippala	1	High School	0.54
Hannah Wang	3	High School	0.53
Amanda Pekau	1	Masters	0.53
Mia Stonov	3	High School	0.41
Sonali Sharma	3	High School	0.35
Umer Jalil	2	Undergraduate	0.32
Rana Abdalla	2	High School	0.28

Education level indicates the highest level of education completed. Raters with education level of High School were enrolled in undergraduate degree programs at the time of rating. Correlation coefficients are Pearson correlations with the average AI score of 10 iterations for each article. Each rater scored 23-24 articles depending on their assigned subgroup.

Additional correlations:
Pearson correlation of median AI score across 10 iterations with median human score: r=0.88
Pearson correlation of first gathered AI score with average human score: r=0.84

Appendix I: Relationship between Google News Article Index Number and Article Sentiment Score

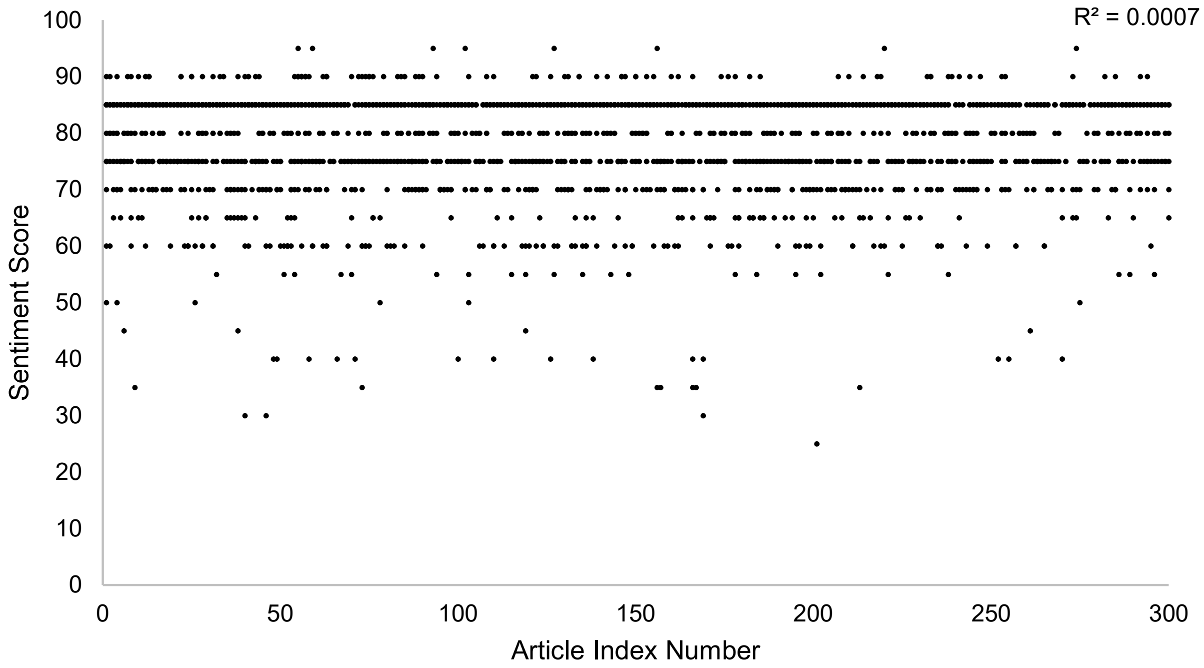

Up to 300 articles were sampled from each calendar year. Article index number represents the order in which the article was presented by the Google News algorithm for a given calendar year, with 1=the first article indexed and 300=the 300th article indexed. All articles with sentiment scores from 2000-2025 (n=2168) are included in the scatter plot.
